# Expanding the Cutaneous Spectrum of Nicolaides‑Baraitser Syndrome: Eczema and Generalized Hair Loss

**DOI:** 10.7759/cureus.88854

**Published:** 2025-07-27

**Authors:** Brian A Moreno, Karina Torres, Ana Duarte

**Affiliations:** 1 Dermatology, Lake Erie College of Osteopathic Medicine, Bradenton, USA; 2 Dermatology, Nicklaus Children's Hospital, Children's Skin Center, Miami, USA

**Keywords:** academic dermatology, clinical dermatology, dermatology case report, general dermatology, genetics medical education, medical dermatology, pediatric dermatology, pediatric genetics, research in dermatology, skin disease/dermatology

## Abstract

Nicolaides‑Baraitser syndrome (NBS) is an ultrarare SMARCA2‑related neurodevelopmental disorder, whose cutaneous hallmarks traditionally include early hypotrichosis and coarse, sparse scalp hair. We describe a four‑year‑old girl with genetically confirmed NBS who presented with years‑long, worsening eczematous dermatitis and diffuse, non‑scarring alopecia that far exceeded the baseline hypotrichosis described in the syndrome. Clinical examination revealed erythematous, lichenified patches distributed across the trunk, extremities, and flexural creases, accompanied by generalized scalp thinning without follicular dropout or scarring. Standard emollient care and low‑ to mid‑potency topical corticosteroids had provided only transient relief; a step‑up regimen was initiated consisting of twice‑daily mometasone cream for flares, tacrolimus 0.03 % ointment for maintenance, wet‑wrap therapy, oral antihistamines, and barrier‑repair moisturizers. Hair‑loss management included nightly topical mometasone solution and ketoconazole shampoo, together with multivitamin supplementation. The constellation of severe atopic dermatitis and progressive alopecia in this patient suggests that SMARCA2 dysfunction may compromise epidermal barrier integrity and hair‑follicle cycling, rendering affected children particularly susceptible to eczematous inflammation and telogen shift. Early dermatologic recognition and aggressive barrier‑focused therapy are therefore pivotal for mitigating pruritus, preventing secondary infection, and improving quality of life in NBS. This case broadens the dermatologic phenotype of NBS and underscores the need for prospective studies to clarify genotype-phenotype correlations and guide targeted interventions for skin and hair involvement in this syndrome.

## Introduction

Nicolaides‑Baraitser syndrome (NBS) is a very infrequent neurodevelopmental condition first delineated in the early 1990s and now known to result from heterozygous de novo missense variants in SMARCA2, which encodes the ATP‑dependent chromatin‑remodeling protein BRM [1,2]. Fewer than 250 molecularly confirmed individuals have been reported, making NBS one of the rarest Mendelian disorders in the SWI/SNF (BAF) complex spectrum [2]. Pathogenic variants cluster within the helicase ATPase domain of SMARCA2 and appear to exert dominant‑negative effects on chromatin remodeling, thereby disrupting transcriptional programs essential for neural, skeletal, and epidermal development [3]. Genotype-phenotype studies show that substitutions affecting helicase motifs V and VI often correlate with more severe intellectual disability, while variants outside these motifs may spare seizure susceptibility, indicating allelic heterogeneity within the syndrome [4].

Clinically, NBS is distinguished by coarse facial features (deep‑set eyes, broad nasal tip, thick vermilion), short stature, brachydactyly with prominent interphalangeal joints, severe speech delay, and seizures in roughly half of patients [1,4]. Cutaneous findings are regarded as early diagnostic clues: infants typically exhibit sparse, wiry scalp hair that remains thin or may even progress to diffuse hypotrichosis over time [1,5]. Xerosis and eczematous plaques have been anecdotally mentioned, but the breadth and severity of inflammatory skin disease in NBS are yet to be systematically characterized [5]. Likewise, hair‑loss patterns beyond congenital hypotrichosis, such as later‑onset diffuse non‑scarring alopecia, have not been formally analyzed.

Here, we describe a four‑year‑old girl with genetically confirmed NBS who developed chronic, treatment‑refractory atopic dermatitis alongside progressive, generalized hair loss. By detailing her dermatologic presentation and management, we aim to broaden the cutaneous phenotype of NBS and highlight the importance of early, multidisciplinary surveillance for skin and hair manifestations in this extremely rare chromatin‑remodeling disorder. Although characteristic facial features and skeletal anomalies are often emphasized in NBS, our patient lacked several hallmark findings, such as coarse facial features and brachydactyly, highlighting the phenotypic variability that can occur even among genetically confirmed cases.

## Case presentation

**Figure 1:**
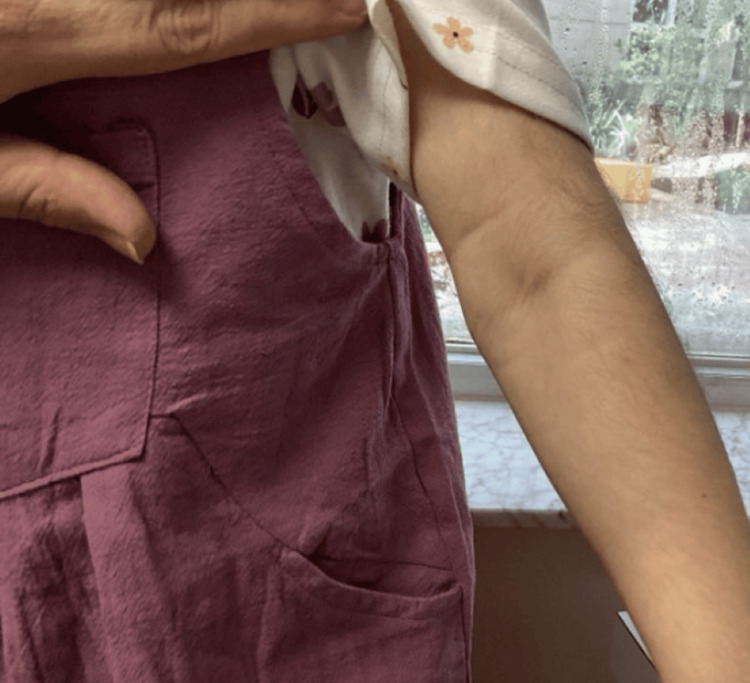
Erythematous, lichenified plaques with fine scale across the left arm, set against markedly xerotic skin

**Figure 2:**
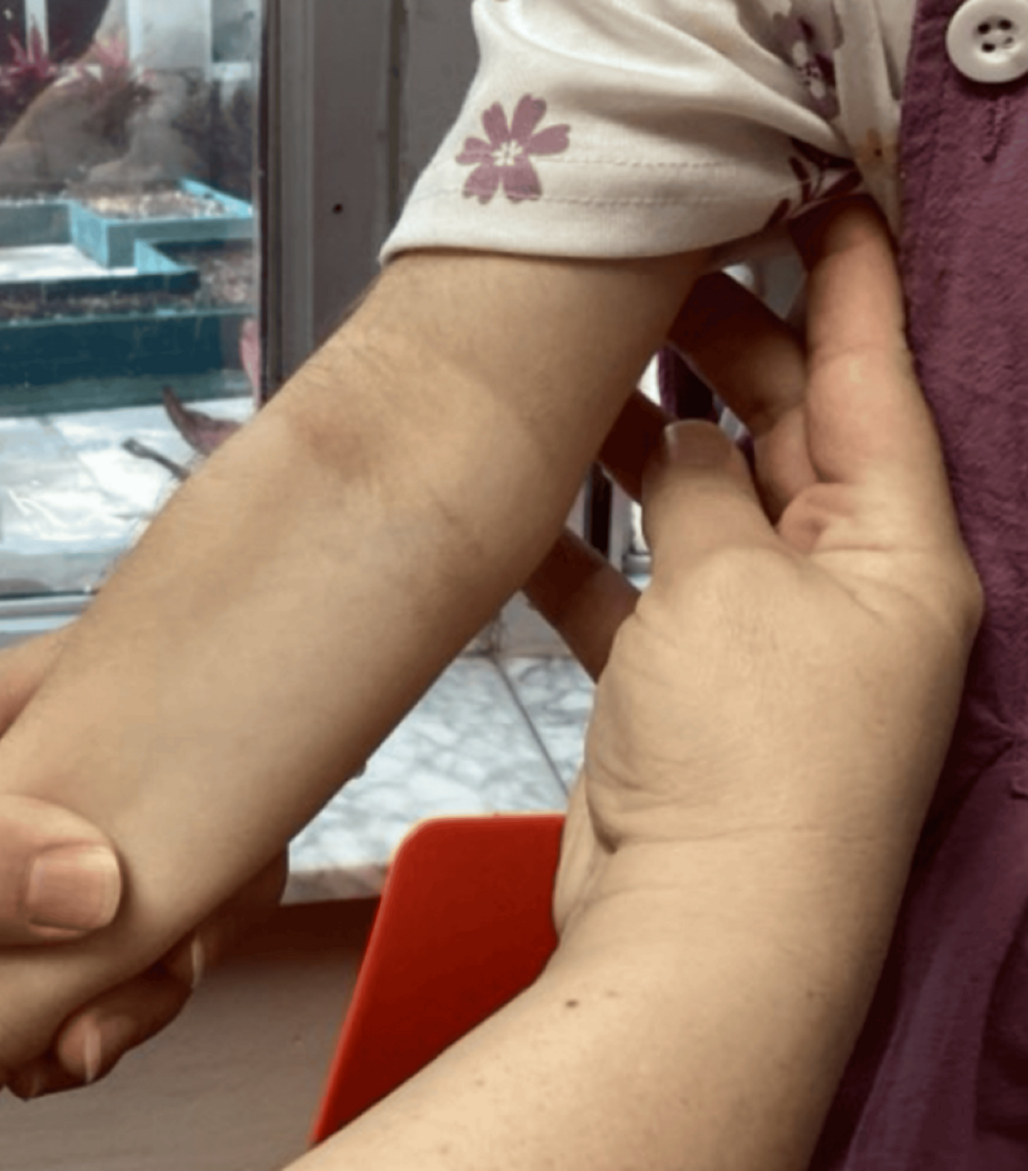
Erythematous, lichenified plaques with fine scale across the right arm, set against markedly xerotic skin

**Figure 3:**
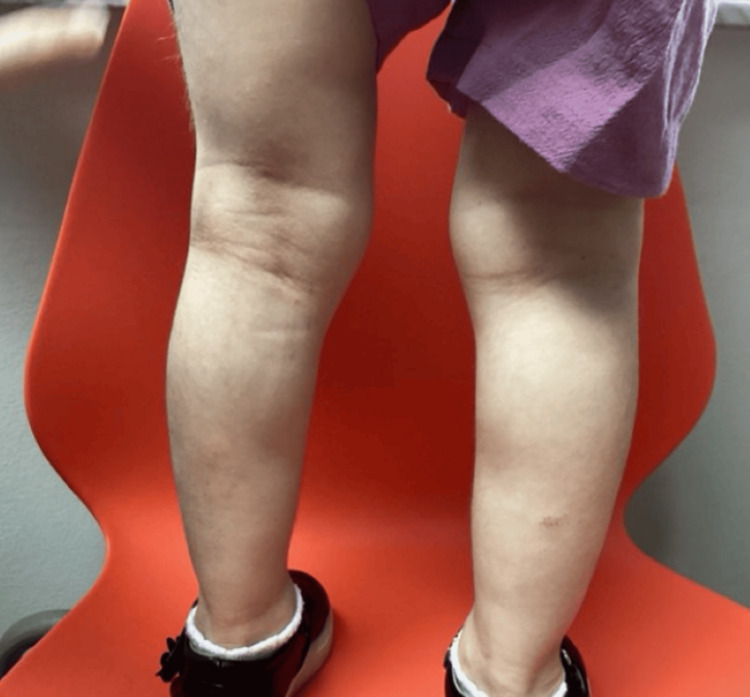
Erythematous, lichenified plaques with fine scale across both legs, set against markedly xerotic skin

**Figure 4:**
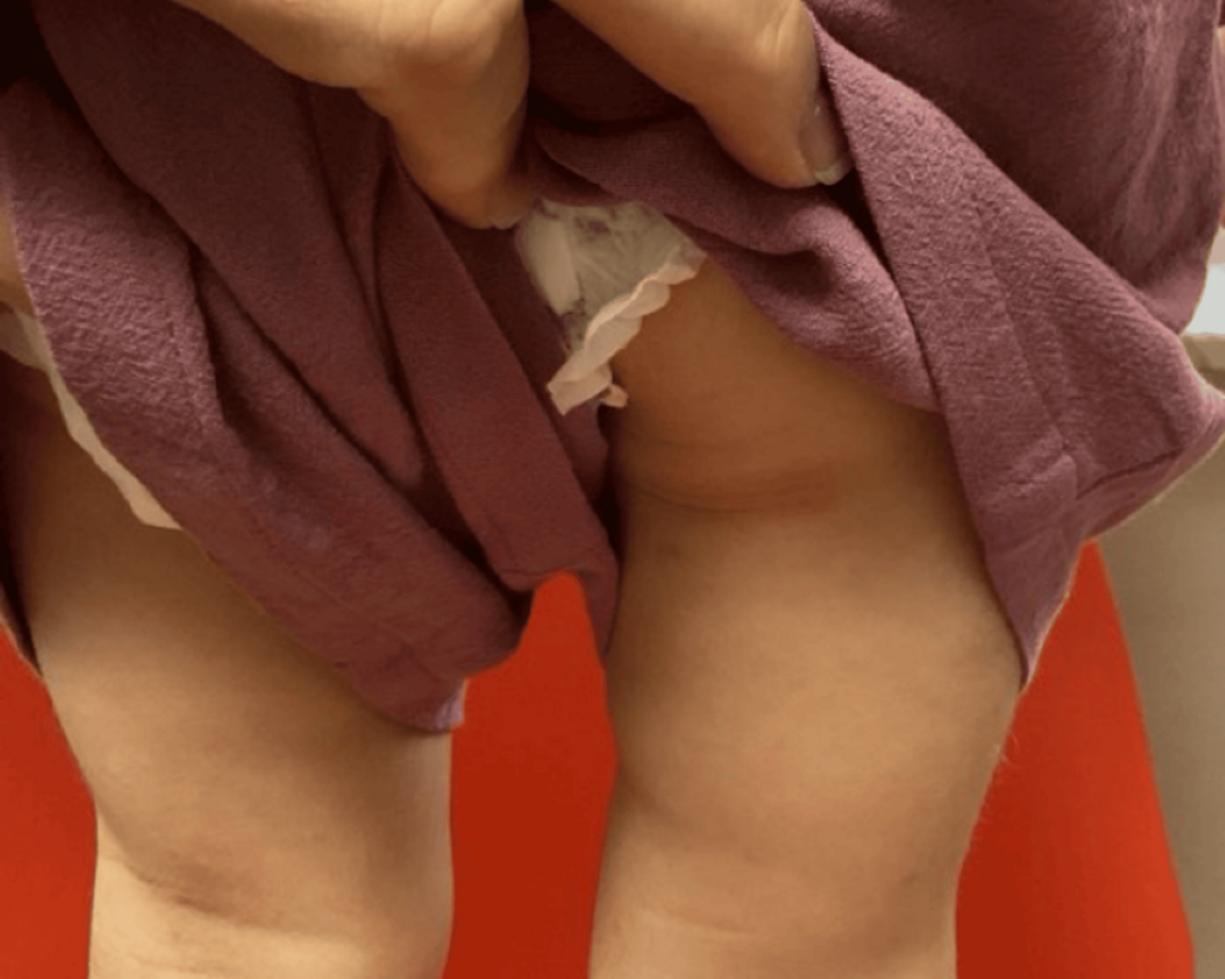
Erythematous, lichenified plaques with fine scale across the left buttock, set against markedly xerotic skin

A four‑year‑old girl with genetically confirmed NBS was seen in our pediatric dermatology clinic for a long‑standing but recently worsening rash and progressive scalp hair loss. Her medical history included developmental delay typical of NBS and bilateral tympanostomy tube placement; she was otherwise healthy and followed by neurology, orthopedics, and otolaryngology. Topical hydrocortisone and fluticasone had been used intermittently for several years, yet her mother reported escalating pruritus, night‑time scratching, and diffuse hair thinning that surpassed the baseline hypotrichosis usually observed in NBS. Dermatologic findings consisted of erythematous, lichenified plaques with fine scale across the flexures and buttocks, set against markedly xerotic skin (Figures 1-4). No crusting or pustulation suggested secondary infection. The scalp showed generalized non‑scarring alopecia with intact follicular ostia and absent perifollicular erythema or scale, while nails and mucosae were unremarkable.

Given the chronic, moderate‑to‑severe atopic dermatitis flare and diffuse alopecia, we intensified barrier and anti‑inflammatory therapy. Mometasone 0.1 % cream was prescribed twice daily to active lesions for up to 14 days, followed by tacrolimus 0.03 % ointment twice daily for maintenance. Parents were instructed to bathe the child with a fragrance‑free hydrating cleanser, apply CeraVe cream or healing ointment at least three times per day, and employ wet‑wrap therapy during flares while avoiding scented detergents, fabric softeners, and perfumed products. Pruritus control consisted of cetirizine 2.5 mg nightly and loratadine 2.5 mg each morning as needed. For the scalp, mometasone 0.1 % solution was ordered nightly and ketoconazole 2 % shampoo twice weekly to mitigate inflammation; a multivitamin with B‑complex and omega‑3 fatty acids was recommended to support hair growth. We counseled the family on the relapsing course of atopic dermatitis, the importance of consistent barrier care, proper steroid tapering, and signs of secondary infection requiring prompt evaluation. A six‑week follow‑up was arranged, with escalation to systemic therapy, such as dupilumab, if topical measures proved inadequate. Unfortunately, the patient did not return to our clinic for follow-up, and her subsequent dermatologic course was monitored by other subspecialists.

## Discussion

NBS is among the rarest Mendelian disorders of the SWI/SNF chromatin‑remodeling complex and is typically recognized by its distinctive facies, intellectual disability, and early‑onset hypotrichosis that yields sparse, wiry scalp hair [1,2]. Large clinical surveys emphasize xerosis as the most frequent cutaneous accompaniment, whereas inflammatory dermatoses have been mentioned only in passing, and no detailed accounts of chronic, lichenified eczema have been published to date [1,5]. The severe, treatment‑refractory atopic dermatitis observed in our patient therefore broadens the dermatologic spectrum of NBS and identifies eczematous inflammation as a potential, under‑recognized driver of morbidity in this population. The concomitant diffuse non‑scarring alopecia likewise exceeds the baseline congenital hypotrichosis ordinarily described, suggesting that active dermatitis and telogen shedding may synergize to accentuate hair loss.

Pathogenic NBS variants cluster within helicase motifs of the SMARCA2 ATPase domain and are thought to exert dominant‑negative effects on SWI/SNF chromatin remodeling, thereby dysregulating gene expression programs critical for neural, skeletal, and epidermal development [3]. Genotype-phenotype studies indicate that substitutions involving motifs V and VI correlate with more severe neurologic impairment and seizures, underscoring functional hot spots within the gene [4]. Although SMARCA2’s role in epidermal differentiation has not been fully elucidated, SWI/SNF complexes govern keratinocyte barrier‑gene transcription; their disruption could plausibly weaken tight‑junction integrity, increase transepidermal water loss, and predispose to atopic dermatitis [3,4]. The coexistence of chronic eczema and pronounced alopecia in our patient lends clinical support to this mechanistic hypothesis and calls for further investigation of skin‑barrier biology in NBS.

Review of published cases highlights how rarely eczema and progressive alopecia have been documented. Among the fewer than 250 molecularly confirmed individuals, reports typically mention “dry skin” or “hypotrichosis” without elaborating on disease severity, therapeutic response, or quality‑of‑life impact [1,5]. Such omissions may reflect neurologic and craniofacial predominance in genetic reports, underreporting of dermatologic findings, or genuine phenotypic heterogeneity modulated by variant position within SMARCA2 [4]. Prospective, dermatology‑focused registries are needed to clarify the prevalence and natural history of inflammatory skin disease, hair‑shaft fragility, and telogen effluvium in NBS. As such, this report provides a hypothesis-generating observation that warrants validation through larger, dermatology-focused studies in the NBS population.

Management of our patient adhered to conventional pediatric atopic‑dermatitis algorithms-mid‑potency topical corticosteroids for flares, calcineurin inhibitors for maintenance, rigorous emollient therapy, antihistamines for pruritus, and anti‑inflammatory scalp care. The partial response observed underscores the importance of early escalation to systemic biologics such as dupilumab when topical regimens fail, particularly because developmental delay and sensory issues can hamper adherence [1]. Comprehensive caregiver education on barrier care, steroid safety, and infection surveillance remains essential.

In summary, this case documents severe atopic dermatitis and diffuse non‑scarring alopecia in a child with NBS, thereby expanding the recognized cutaneous phenotype and suggesting that chromatin‑remodeling defects may compromise skin‑barrier integrity and hair‑follicle cycling. Early dermatologic referral, aggressive barrier‑focused therapy, and multidisciplinary follow‑up are imperative for optimizing outcomes in this ultrarare condition. Future studies integrating detailed skin assessments with molecular data will be critical for delineating genotype‑dependent dermatologic risk and guiding targeted interventions for patients with NBS.

## Conclusions

This case highlights severe, chronic atopic dermatitis and diffuse non‑scarring alopecia as previously under‑recognized manifestations of NBS. The findings suggest that SMARCA2‑driven chromatin‑remodeling defects may compromise epidermal barrier integrity and hair‑follicle cycling, predisposing affected children to inflammatory skin disease and progressive telogen shedding. Early dermatologic evaluation, aggressive barrier‑focused therapy, and readiness to escalate to systemic treatments are essential for minimizing morbidity and improving quality of life in this ultrarare population. As this report describes a single case, findings should be considered preliminary and hypothesis-generating. Prospective, genotype-linked dermatology registries will be crucial for defining the true prevalence of eczema and alopecia in NBS and for informing targeted therapeutic strategies.

## References

[1] Abdul-Rahman O, May F (1993-2025). SMARCA2-related Nicolaides-Baraitser syndrome. GeneReviews® (Internet).

[2] (2025). MedlinePlus. Nicolaides-Baraitser syndrome. https://medlineplus.gov/genetics/condition/nicolaides-baraitser-syndrome/.

[3] Castori M, Covaciu C, Rinaldi R, Grammatico P, Paradisi M (2008). A rare cause of syndromic hypotrichosis: Nicolaides-Baraitser syndrome. J Am Acad Dermatol.

[4] Sousa SB, Hennekam RC (2014). Phenotype and genotype in Nicolaides-Baraitser syndrome. Am J Med Genet C Semin Med Genet.

[5] Sousa SB, Abdul-Rahman OA, Bottani A (2009). Nicolaides-Baraitser syndrome: Delineation of the phenotype. Am J Med Genet A.

